# A ‘Split-Gene’ Transketolase From the Hyper-Thermophilic Bacterium *Carboxydothermus hydrogenoformans*: Structure and Biochemical Characterization

**DOI:** 10.3389/fmicb.2020.592353

**Published:** 2020-10-30

**Authors:** Paul James, Michail N. Isupov, Simone Antonio De Rose, Christopher Sayer, Isobel S. Cole, Jennifer A. Littlechild

**Affiliations:** Henry Wellcome Building for Biocatalysis, Biosciences, University of Exeter, Exeter, United Kingdom

**Keywords:** hyperthermophilic, ‘split-gene’, transketolase, thermal stability, industrial applications

## Abstract

A novel transketolase has been reconstituted from two separate polypeptide chains encoded by a ‘split-gene’ identified in the genome of the hyperthermophilic bacterium, *Carboxydothermus hydrogenoformans*. The reconstituted active α_2_β_2_ tetrameric enzyme has been biochemically characterized and its activity has been determined using a range of aldehydes including glycolaldehyde, phenylacetaldehyde and cyclohexanecarboxaldehyde as the ketol acceptor and hydroxypyruvate as the donor. This reaction proceeds to near 100% completion due to the release of the product carbon dioxide and can be used for the synthesis of a range of sugars of interest to the pharmaceutical industry. This novel reconstituted transketolase is thermally stable with no loss of activity after incubation for 1 h at 70°C and is stable after 1 h incubation with 50% of the organic solvents methanol, ethanol, isopropanol, DMSO, acetonitrile and acetone. The X-ray structure of the holo reconstituted α_2_β_2_ tetrameric transketolase has been determined to 1.4 Å resolution. In addition, the structure of an inactive tetrameric β_4_ protein has been determined to 1.9 Å resolution. The structure of the active reconstituted α_2_β_2_ enzyme has been compared to the structures of related enzymes; the E1 component of the pyruvate dehydrogenase complex and D-xylulose-5-phosphate synthase, in an attempt to rationalize differences in structure and substrate specificity between these enzymes. This is the first example of a reconstituted ‘split-gene’ transketolase to be biochemically and structurally characterized allowing its potential for industrial biocatalysis to be evaluated.

## Introduction

Transketolase (TK, EC 2.2.1.1) is a thiamine diphosphate-dependant (TPP) enzyme which plays an important role in the pentose phosphate pathway. It catalyses the rearrangement of sugar molecules by the transfer of a C2 unit from D-xylulose-5-phosphate to erythrose-4-phosphate, resulting in the formation of fructose-6-phosphate and glyceraldehyde-3-phosphate which are fed back into the glycolysis pathway (Racker et al., 1954).

**SCHEME 1:**
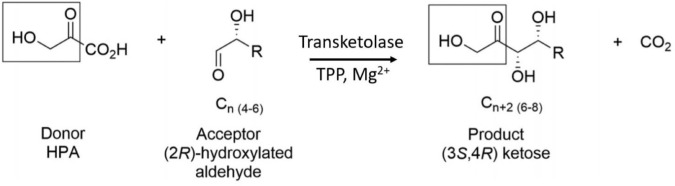
TK catalyzed conversion of hydroxypyruvate and an acceptor aldehyde to a ketose product and carbon dioxide.

TK enzymes have been found to accept a broad range of donor and acceptor substrates including xylulose 5-phosphate, ribose 5-phosphate, fructose 6-phosphate, glyceraldehyde 3-phosphate, erythrose 4-phosphate and hydroxypyruvate (HPA) (Schenk et al., 1998). Use of HPA as the ketol donor allows the release of the volatile reaction product CO_2_ which drives the reaction to completion (Scheme 1). The ability of TKs to form enantioselective carbon-carbon bonds has generated increasing interest for their use as biocatalysts in industrial synthetic reactions (Hibbert et al., 2008). This reaction has previously been described as irreversible due to the production and evolution of CO_2_ but was shown by Marsden et al. (2017) to be reversible over a period of weeks because while the decarboxylation of hydroxypyruvate is virtually irreversible, the carbon-carbon bond formation is not. The *Escherichia coli* TK (EcTK) has previously been used to demonstrate the potential synthesis of a range of pharmaceutical relevant sugars with HPA as the ketol donor and glycolaldehyde as the acceptor to produce the sugar erythrulose at a 100% conversion (Lilly et al., 1996).

TK enzymes are found throughout nature and have been isolated and structurally characterized from a number of different organisms including *E. coli* (Littlechild et al., 1995; Martin, 2008; Ludtke et al., 2013), *Thermus thermophilus* (PDB: 2E6K), *Saccharomyces cerevisiae* (Lindqvist et al., 1992; Nikkola et al., 1994), maize (Gerhardt et al., 2003), *Leishmania mexicana* (Veitch et al., 2004), *Mycobacterium tuberculosis* (Fullam et al., 2012), and human (Mitschke et al., 2010). Most TKs have a monomeric molecular mass ∼70 kDa and are active as homodimers with amino acid residues from each monomer contributing to the two active sites. Each of these active sites contains a molecule of TPP and a Mg^2+^ or Ca^2+^ ion which plays an important role in the co-factor binding (Selivanov et al., 2003).

TPP dependent enzymes are similar in that they all bind the cofactor at the interface of the two domains of each monomer: the pyrophosphate binding domain (PP) and the pyrimidine binding (Pyr) domain (Muller et al., 1993). The TK-like enzymes are a subgroup of homo-dimeric TPP enzymes which contain the N-terminal PP domain, followed by the Pyr domain and a transketolase C-terminal domain (TKC). The TKC domain is thought to have been introduced along the evolutionary route (Costelloe et al., 2008). The TK-like group contains related enzymes such as, D-xylulose-5-phosphate synthase (DXPS) and the E1 component of the large multi-subunit pyruvate dehydrogenase (PDH). Both DXPS and bacterial PDH E1 have the same order of domains (PP-, Pyr-, TKC) along the polypeptide chain (Xiang et al., 2007). Although the DXPS and PDHs E1 have limited sequence identity to TKs (around 26–30%) the arrangement of domains within the dimer and TPP binding sites of these enzymes are very similar (Muller et al., 1993; Arjunan et al., 2002). The major difference between TK and these related enzymes is that the TPP binds on the interface of the PP domain of one monomer and the Pyr domain of the adjacent one to form the TK dimer whereas in DXPS and bacterial PDH E1 the active sites are formed between the PP and Pyr domains within the same subunit (Costelloe et al., 2008).

Interestingly the mammalian PDH E1 (Kato et al., 2008) component proteins are hetero-tetramers with mutual positions of domains quite similar to those observed in bacterial dimeric PDHs, however they are built up from two polypeptide chains of comparable size with the PP domain located on one chain and the Pyr and the TKC domains on the second chain.

It has been previously reported that many archaea are lacking some, if not all of the enzymes, that constitute the pentose phosphate pathway, and the TK enzyme is either absent in the archaeal genomes or is encoded by two separate genes, referred to as a ‘split-gene’, which may or may not be located next to each other on the genome (Bräsen et al., 2014). The thermophilic and hyper-thermophilic bacteria have been reported to contain either a full-length or both a full-length and ‘split-gene’ TK enzymes, where the latter has been proposed to be acquired by horizontal gene transfer from the archaea (Koonin and Galperin, 2013).

To date only a small number of thermostable TK enzymes have been biochemically characterized. The TK from *Geobacillus stearothermophilus* (GsTK) has been shown to have an optimum temperature of 70°C and has the ability to use a range of aldehydes as the acceptor in the potential commercial reaction with hydroxypyruvate as the ketol donor (Abdoul-Zabar et al., 2013). Also characterized are two TK enzymes from *Deinococcus geothermalis* (DgTK) and *Deinococcus radiodurans* (DrTK) that were shown to have an optimal temperature of 50°C and have been used in combination with a thermostable transaminase enzyme to produce L-gluco-heptulose from L-arabinose (Bawn et al., 2018). The TK from the thermophilic bacterium *T. thermophilus* has provided structural insights that have been used to guide the design of mutants of the *E. coli* TK (EcTK) to increase its thermal stability (Morris et al., 2016).

*Carboxydothermus hydrogenoformans* Z-2901 is a hyper-thermophilic, anaerobic bacterium isolated from a hot swamp of Kunashir Island, Russia (Svetlichny et al., 1991). This organism has an optimal growth temperature of 78°C and is believed to be one of the fastest growing carbon monoxide (CO) utilizing bacteria known and has five highly differentiated anaerobic CO dehydrogenase complexes (Wu et al., 2005).

Here, we report the biochemical and structural characterization of the ‘split-gene’ TK from this organism (ChTK-F) which has been reconstituted by combining the two proteins encoded on the *C. hydroxydothermus* genome. This is the first example of a ‘split-gene’ TK that has been reconstituted and found to be active. This enzyme has been biochemically and structurally characterized which has allowed its potential application for industrial biocatalysis to be evaluated.

## Results and Discussion

### Gene Identification, Protein Expression, and Purification

The search for a TK enzyme in thermophilic bacteria using the BLAST database with the EcTK as the query sequence revealed the genes ChTK-N (Accession number: ABB15544) and ChTK-C (Accession number: ABB16214) in the bacterium *C. hydrogenoformans.* These genes which make up the reconstituted ChTK-F have 32% sequence identity to the EcTK (PDB: 1QGD) and 40% sequence identity to the human TK (PDB: 3MOS). The ChTK-F also has 27% sequence identity to the DXPS from *E. coli* (PDB: 2O1S) and 27% sequence identity to the E1 subunit of the multi-subunit PDH from *E. coli* (PDB: 1L8A).

**FIGURE 1:**
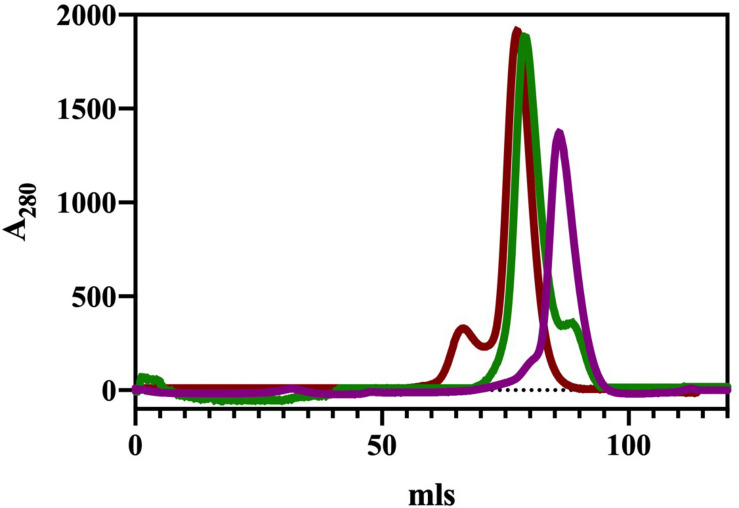
Gel filtration chromatography elution (Superdex 200) of ChTK-C, ChTK-N and ChTK-F. ChTK-C with apparent molecular mass suggesting a tetramer (∼132 kDa) (red); ChTK-N was eluted as a dimer (∼62 kDa) (purple); and ChTK-F was eluted as a heterotetramer (green). A slight decrease in the MW can be observed between the ChTK-F and ChTK-C peak as well as a small ChTK-N peak in the ChTK-F trace corresponding to the excess ChTK-N used in the reaction.

The ChTK-N and ChTK-C genes were successfully cloned into the pLATE51 (Thermo Scientific) expression vector and over expressed in *E. coli* BL21 DE3^∗^ using the auto induction ZYM-5052 medium (Studier, 2005). After purification by Ni-NTA affinity chromatography and size exclusion chromatography (GF-200) the purified recombinant proteins ran as single bands on SDS-PAGE at the calculated molecular mass of the His-tagged proteins (31.0 kDa and 33.2 kDa). The apparent molecular mass of the native enzyme after gel filtration chromatography indicated that ChTK-N eluted as a dimer (∼62 kDa) whereas ChTK-C was purified as a tetramer (∼132 kDa) (Figure 1). The active ChTK-F was reconstituted by mixing the two proteins ChTK-N (dimer) and ChTK-C (tetramer) at 4°C overnight (with an excess of ChTK-N) and was purified by size exclusion chromatography. The formation of the complex was analyzed using size exclusion chromatography (GF-200) (Figure 1) in which a slight decrease in MW can be observed between the ChTK-F and ChTK-C peaks as well as small ChTK-N peak corresponding to the excess ChTK-N used in the reaction. The purity of the collected ChTK-F fraction was sufficient for crystallization of the complex.

### A ‘Split-Gene’ TK

TK enzymes are widespread in nature with most eukaryotic and bacterial genomes containing at least one TK gene encoding a single protein of 600–700 amino acids. The presence of a full-length or ‘split-gene’ TK in some bacterial and archaeal genomes has been investigated using a BLAST search with the EcTK or ChTK protein sequence as a reference. Previous studies have reported that archaea are either missing the TK enzyme altogether or the enzyme is encoded by two different genes that can either be situated next to each other on the genome (*Sulfolobus solfataricus)* (She et al., 2001) or can be spread across the genome (*Methancaldococcus jannaschii*) (Soderberg, 2005). The thermophilic and hyper-thermophilic bacteria have been reported to possess either both the ‘split-gene’ and the full-length TK enzyme (*Thermotoga maritima*) (Rodionova et al., 2012) or just the full-length TK enzyme (*G. stearothermophilus*) (Egan et al., 2017). Mesophilic bacteria seem to only have the full-length TK enzyme (*E. coli*, *Lactobacillus plantarum*). However, the *C. hydrogenoformans* has been found in this study to not contain a full-length TK encoding gene and to only code for a ‘split-gene’ which is novel for any organism outside of the archaeal kingdom. The genome of the hyperthermophilic archaeon *Nanoarchaeum equitans* contains numerous examples of ‘split-gene’ proteins that are encoded by single genes in other archaea and it has been suggested that multi-domain proteins such as TK might have evolved from the fusion of these different ‘split-genes’. It has been proposed that the presence of such ‘split-genes’ in a microorganism could reflect its ancestral state (Waters et al., 2003) and also be involved in the evolutionary path of new enzymes.

### Enzyme Activity

Initial activity measurements of the gene products ChTK-N and ChTK-C and reconstituted enzyme, ChTK-F were carried out using a colorimetric tetrazolium red assay (Smith et al., 2006) that showed that the individual ChTK-N and ChTK-C did not possess TK activity but the reconstituted ChTK-F was active. This activity was confirmed using a more accurate HPLC assay (Supplementary Figure S1) and this subsequently has led to the ChTK-F to be biochemically characterized.

Using the substrate glycolaldehyde as the aldehyde acceptor and HPA (100 mM) as the ketol donor the ChTK-F was found to have a Km of 41 ± 4 mM and a kcat/Km of 0.25 ± 0.01 s^–1^ mM^–1^. These kinetic constants show that the enzyme has a higher Km and lower kcat/Km toward these substrates in comparison to both the yeast and EcTK enzyme (Yi et al., 2012) which is expected for a hyperthermophilic enzyme assayed at room temperature. The kinetic data is similar to that previously reported for a mutant EcTK (D469E) enzyme (Yi et al., 2012) but this amino acid substitution is not present in the ChTK-F enzyme. The reconstituted ChTK-F enzyme was assayed using a range of aldehyde acceptors and was shown to be active toward the bulkier substrates phenylacetaldehyde and cyclohexanecarboxaldehyde (Supplementary Figure S5). EcTK has been shown to have some activity toward these substrates however the yields were low and by-products were formed, which was attributed to the steric hindrance for binding of these bulkier substrates in the enzyme active site (Hobbs et al., 1993; Morris et al., 1996).

### Temperature and pH Stability

**FIGURE 2:**
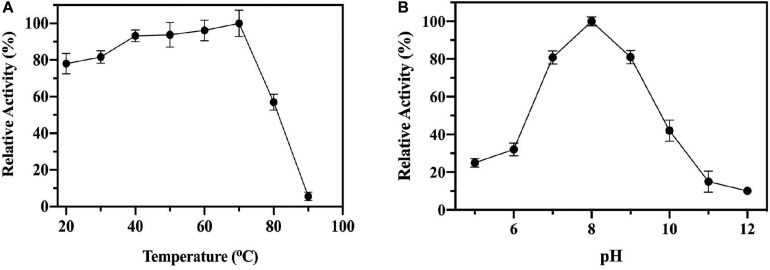
The biochemical characterization of ChTK-F. **(A)** The percentage of relative enzyme activity for ChTK-F after incubation for 1 h at a range of temperatures indicated against a control kept at room temperature in a reaction with HPA as a ketol donor and glycolaldehyde as an acceptor. **(B)** The percentage of relative activity for ChTK-F after incubation for 1 h at different pHs.

To test the enzyme stability and activity at elevated temperatures the ChTK-F enzyme was incubated at increasing temperatures for 1 hour and then cooled to room temperature before being assayed. No loss of activity was observed for the ChTK-F after incubation for 1 hour at temperatures up to 70°C. The enzyme activity was reduced to ∼50% after incubation for 1 hour at 80°C and became denatured with a complete loss of activity after incubation at 90°C (Figure 2A), This is a small improvement in stability as measured from these experiments from that reported with the on the GsTK enzyme (Abdoul-Zabar et al., 2013).

Thermally stable proteins are often able to withstand a number of other denaturing conditions such as extremes of pH. The pH stability of the ChTK-F protein was tested by incubation for one hour in the range pH 5.0 – 12.0 (Figure 2B). The enzyme showed the highest activity at pH 8.0 and was able to retain ∼ 50% activity after incubation at pH 10. The ChTK-F pH at which the enzyme retained maximum activity is similar to that reported in the literature for the TK from the moderate thermophile *G. stearothermophilus*, pH 7.0–8.0 (Abdoul-Zabar et al., 2013), *D. geothermalis*, pH 8.0 (Bawn et al., 2018) and the mesophilic EcTK, pH 8.0–8.5 (Littlechild et al., 1995; Martin, 2008).

### Solvent Stability

**FIGURE 3:**
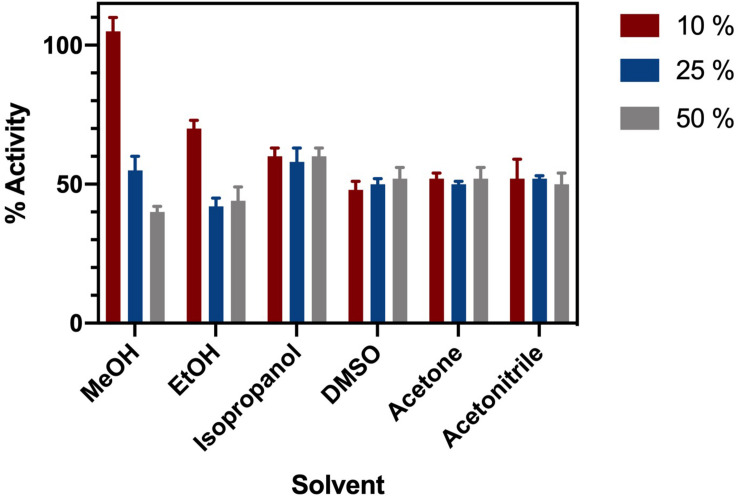
Solvent Stability of ChTK-F. The percentage of relative enzyme activity for ChTK-F after incubation for 1 h in buffer and with 10% (Black Bar), 25% (Blue Bar), and 50% (Yellow Bar) of the organic solvents methanol, ethanol, isopropanol, DMSO, acetonitrile and acetone.

ChTK-F was incubated in a range of common organic solvents and its residual activity measured (Figure 3). The enzyme retained ∼ 60% activity after incubation for 1 hour in buffer containing either 10%, 25% or 50% methanol, ethanol, isopropanol, DMSO, acetonitrile and acetone. The lowest enzyme activity was in 25 – 50% ethanol where it retained ∼ 40% of its relative activity compared to the control. The final concentration of solvent in the enzyme assay was no greater than 7.5%. Such solvent stability agrees with the higher thermal stability of ChTK-F.

### Crystallization and Structural Determination

Initial attempts to crystallize the reconstituted active fraction of ChTK-F resulted in crystals of a tetrameric ChTK-C. It appeared that 200 mM ammonium sulfate present in the crystallization conditions had disrupted the formation of the ChTK-F heterotetramer. Later crystallization trials using PEG based conditions and an excess of TPP and Ca^2+^ ions resulted in crystals of the ChTK-F α2β2 protein. These crystals belonged to the space group I222 with cell dimensions of *a* = 123.0, *b* = 130.0, *c* = 165.9 Å and diffracted to 2.1 Å resolution and contained TPP at partial occupancy. Changing the precipitant to malic acid (2.1 M DL-malic acid pH 7.0) produced crystals in the same space group but which diffracted to an improved resolution of 1.4 Å and had full occupancy of the TPP cofactor in the active site.

**FIGURE 4:**
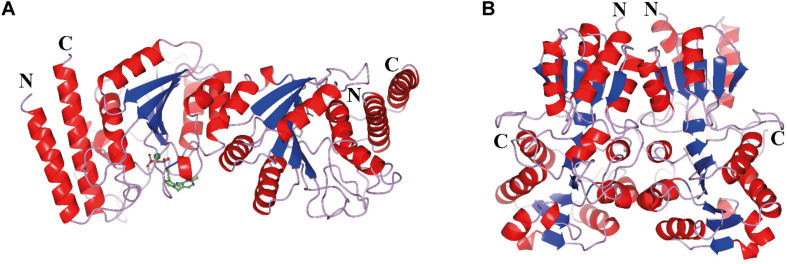
The oligomeric state of the different ChTK polypeptides. **(A)** The modeled ChTK-N dimer is shown as a cartoon model with the N- and C-termini indicated. The catalytic Ca^2+^ is shown as a green sphere and the TPP cofactor as a stick model. **(B)** The ChTK-C homotetramer as determined by X-ray crystallography is shown as a cartoon model with N- and C termini indicated. The helices are shown in red, the strands are shown in blue and the loops in lilac. The Figures 4–7B,C, 8 were prepared using CCP4mg (McNicholas et al., 2011).

**FIGURE 5:**
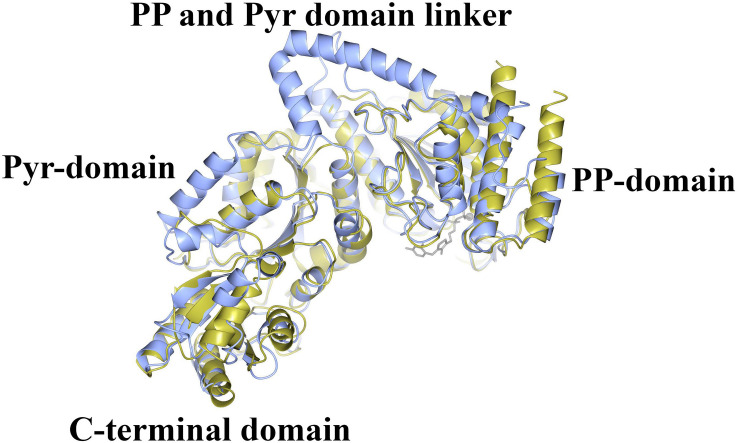
A comparison of the structure of ChTK αβ heterodimer (gold) superimposed onto the monomer of EcTK (ice blue) showing the equivalent positions of the PP-domain, the Pyr-domain and the C-terminal domain. The linker between the PP and Pyr domains is present in EcTK but not in ChTK-F.

The CHTK-F structure shows that two ChTK-N subunits (Figure 4A) and two ChTK-C subunits (Figure 4B) had come together to form a structure similar to the dimeric structure seen for full length transketolases such as EcTK (PDB 1QGD) (Littlechild et al., 1995; Martin, 2008). The division of the ChTK-F heterotetramer into polypeptide chains resembles the structure of the mammalian PDH heterotetramer. As other TK-like group enzymes the ChTK-F is formed by 3 domains of the α/β type, the PP-domain (ChTK-N), the Pyr-domain (ChTK-C residues 1–162) and the C-terminal domain (ChTK-C residues 164–312). The PP and Pyr domains are usually connected by a long flexible linker region in other transketolases and much shorter linkers in DXPSs and bacterial PDHs, however in the ChTK-F structure the linker between the PP and Pyr domain is missing. The linker between the Pyr and the C- terminal domain remains intact (Figure 5). These features potentially allow more flexibility in the reconstituted ChTK-F structure than seen for other full length TKs.

### Hetero-Tetrameric Structure

**FIGURE 6:**
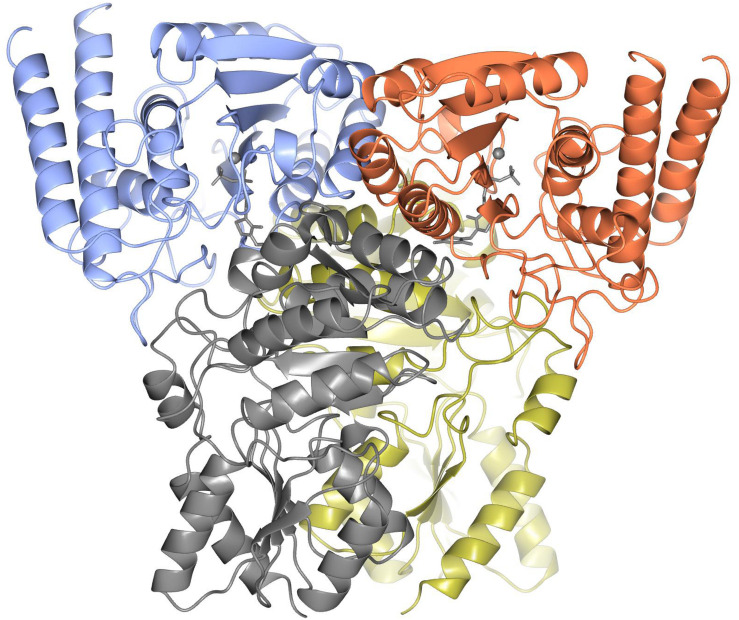
The overall structure of ChTK-F heterotetramer is shown as a cartoon model with the ChTK-N monomers shown in blue and red and the ChTK-C monomers shown in gray and gold with the Ca^2+^ and TPP cofactor shown as stick models in gray.

The ‘split-gene’ ChTK-F protein forms a heterotetramer with a ChTK-C dimer and a ChTK-N dimer coming together to form a similar overall arrangement to that observed in full length TK and TK-like group enzymes (Figure 6). The overall shape of the ChTK-F tetramer can be represented as a trigonal prism with the dimensions 98 × 86 × 76 Å. The heterotetramer has a solvent accessible area of 36780 Å^2^, the formation of the tetramer (in the presence of TPP) buries 17270 Å^2^ or around 30% of the monomer solvent accessible area. However, in the absence of ChTK-C and TPP molecules, the interaction area between the two ChTK-N monomers is limited to around 800 Å^2^ per monomer (6% of the ChTK-N monomer solvent accessible area) and the free ChTK-N in solution is likely to be in equilibrium between a monomer and a dimer (observed at high concentration of protein in size-exclusion chromatography). The ChTK-C dimer formation buries 1700 Å^2^ per monomer (13% of its solvent accessible area), which suggests a dimer is a likely oligomeric form of ChTK-C in solution. The ChTK-C dimer has exposed surface hydrophobic patches on the interface with TPP and ChTK-N. Formation of the ChTK-C homotetramer observed in two crystal forms buries these hydrophobic patches, however only around 5% of each ChTK-C surface accessible area is buried. Such a tetramer is observed in the two crystal forms and in size exclusion chromatography, however it is unlikely to be stable at the lower concentrations of the protein in solution.

### Active Site

**FIGURE 7:**
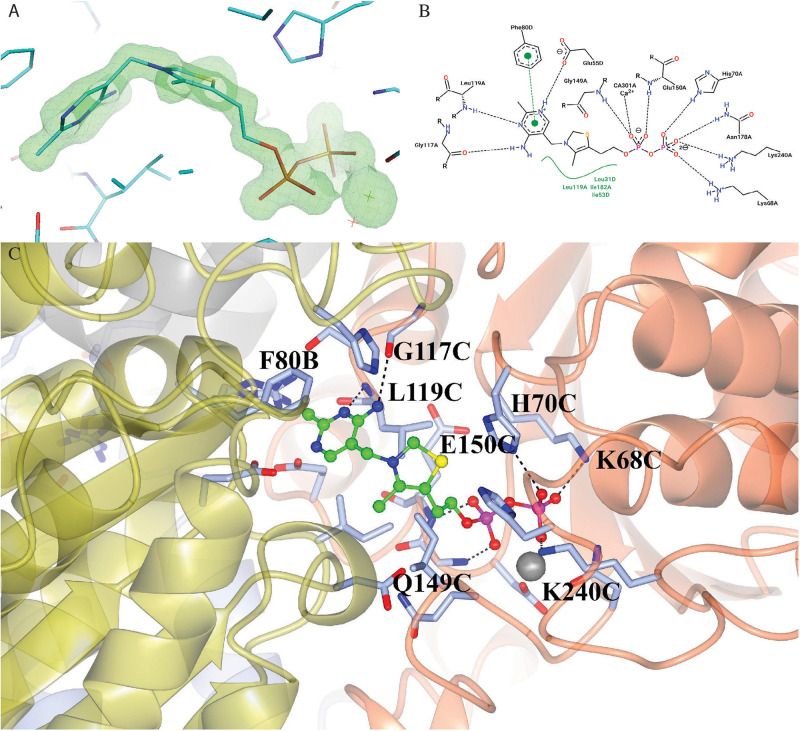
**(A)** Electron- density map showing the TPP and Ca^2+^ ligands bound in the active site of ChTK-F. The weighted F_0_-F_*c*_ omit map, where ligands where not used in the phase calculations, is shown in green and is contoured at 5.0 sigma. The neighboring residues are shown as stick models and the Ca^2+^ ion is shown as a green star. The figure was prepared using the PyMOL Molecular Graphics System, Version 2.0 Schrödinger, LLC. **(B)** A 2-D representation of the TPP binding site between ChTK-N (Chain-A) and ChTK-C (Chain-D). Hydrogen, ion and metal interactions between protein and ligands are drawn as dashed lines. Hydrophobic contacts are represented indirectly by spline section highlighting of the hydrophobic part of the ligand and the label of the contacting amino acid. **(C)** A cartoon model of the TPP and Ca^2+^ binding site between ChTK-N (ice-blue) and ChTK-C (gray) subunits. The TPP molecule is shown as a ball and stick model with carbon atoms colored in green. Amino acid side chains of residues implicated in ligand binding are shown stick models with the hydrogen bonds as black dashes. The Ca^2+^ ion is shown as a gray sphere with its interactions with the TPP molecule and amino acid side chains as red dashes.

The enzyme contains two identical active sites that contain the cofactor TPP and a Ca^2+^ ion bound between a subunit of ChTK-C and its corresponding ChTK-N. The TPP molecule has three moieties, a diphosphate group, a thiazolium ring and an amino-pyrimidine ring and adopts the higher energy V-conformation present in most TPP-dependant protein structures (Figure 7A) (Leeper et al., 2005). The diphosphate group is held in place by a number of hydrogen bonds formed with residues of the CHTK-N monomer (Lys68, His70, Gly149, Glu150, Asn178 and Lys240). The Ca^2+^ ion is held in place by interactions with residues Asp148, Asn178, Leu180 and the oxygen atoms on the diphosphate group. The thiazolium ring is held in place by hydrophobic contacts from both the ChTK-C and ChTK-N monomers (Leu31C, Ile53C, Leu119N and Ile182N). The amino-pyrimidine ring is held in place by another series of hydrogen bonds from both ChTK-C and ChTK-N (Glu55C, Gly117N and Leu119N) as well as a π-π stacking interaction between the pyrimidine ring and Phe80 from ChTK-C (Figures 7B,C).

Previous site-directed mutagenesis studies of the EcTK have shown it is possible to change the substrate specificity of this enzyme to accept bulky aromatic substrates (Payongsri et al., 2012; Payongsri et al., 2015). These studies have shown that mutations at R358, S385, D469, and R520 (EcTK numbering) improve the enzyme activity and yield toward aromatic aldehyde substrates (Supplementary Figure S2). A study by Saravanan et al. (2017b) also attempted to improve the GsTK enzymes activity toward aryl substrates and found another residue L382 (EcTK numbering) was also important for aromatic substrate binding. As none of the amino acids substitutions are present in the ChTK-F enzyme they cannot explain the substrate specificity. A computational docking approach was used to investigate potential the amino acid residues involved in substrate binding in the ChTK-F reconstituted enzyme. Docking studies were performed using Autodock (Kumar et al., 2018) to further rationalize the substrate specificity of the ChTK-F toward bulkier aldehyde substrates. The docking studies revealed a binding orientation of the phenylacetaldehyde substrate in the enzyme active site that places the aromatic ring of this substrate in a position where it could be stabilized by a cation- π interaction with a positively charged lysine residue (K313 ChTK-F). This interaction could not occur in the EcTK as there is a proline residue at this position. The docking suggested that the other residues in ChTK-F that interact with phenylacetaldehyde in the model obtained are in a similar position as in EcTK (Supplementary Figure S3).

Like other TK enzymes ChTK-F has a broad substrate specificity but was found not to accept pyruvate as a ketol donor. This is probably because hydroxypyruvate requires a lower energy of activation for the two-carbon unit transfer in comparison to pyruvate. We know that other TK-like enzymes (such as DXPS) are capable of activating the pyruvate molecule as a two-carbon unit donor but are only able to catalyze a reaction with a single sugar acceptor.

### Structural Basis for Thermostability

The structural determination of the ‘split-gene’ ChTK-F has allowed further insight into the thermal stability of this enzyme when compared to the mesophilic EcTK. ChTK-F has been shown in this study to retain up to 50% activity when heated at 80°C for 1 hour while the EcTK enzyme loses most of its activity at 60°C (Jahromi et al., 2011) and the GsTK enzyme loses activity after 10 min at 75°C (Abdoul-Zabar et al., 2013). A number of structural features are thought to confer thermostability to proteins and these include higher number and clustering of salt bridges, the shortening of surface loops and an increase in hydrophobicity at domain and monomer interfaces (Littlechild et al., 2007, 2013). Previous studies have shown that the TPP cofactor binding plays an important role in preventing deactivation and aggregation of the EcTK at extreme pH, temperature and in the presence of organic solvents (Dalby et al., 2007; Martinez-Torres et al., 2007; Jahromi et al., 2011). This TPP binding is controlled by the formation of two cofactor binding loops containing residues 185–192 and 382–392 (EcTK numbering which correspond to 178–185 and 311–321 in ChTK-F numbering). Attempts have been made to make the EcTK more thermostable by engineering these loops to resemble their equivalent loops of the TK from *T. thermophilus* (TtTK, PDB: 2E6K, 32% identity). In the first cofactor loop Morris et al. (1996) made two mutations (G186R and H192P). The most beneficial of these was H192P which not only increased the activity at 25°C but also increased the Topt to 60°C and retained more activity after heating at 60°C for 1 hour (Morris et al., 2016). This proline residue is conserved in the thermophilic ChTK-F enzyme and is located structurally in the same position as the TtTK enzyme (Supplementary Figure S2). Many thermophilic organisms, usually those with a high GC content in their DNA such as *T. thermophilus* contain more proline residues in the loop regions and these can contribute to high thermostability of the proteins (Suzuki et al., 1987). A comparison reveals that the EcTK and ChTK-F have the same number of prolines (30) compared to the higher number in TtTK (50). While this proline residue clearly plays a role in the enzyme performance at higher temperatures it is a combination of a number of factors that leads to ChTK-F stability and activity at extreme conditions.

### Comparison to Other TPP Containing Enzymes

The ChTK-F enzyme was compared to the DXPS protein sequences from various organisms in an attempt to identify mutations that will allow the enzyme to use pyruvate as a substrate instead of the more expensive hydroxypyruvate. The sequence alignment of various TK and DXPS sequences show a high sequence homology in the residues shown to be involved in donor substrate binding (H66, H100, G114 and H473- EcTK numbering (Supplementary Figure S2). The exception to this is the TK His100 (EcTK numbering) residue which is consistently replaced by phenylalanine in DXPS (Supplementary Figure S4). While the mutation H100F in GsTK increased its activity toward pyruvate (1/10th that of DXPS) the mutation H100L increased it even further (1/3rd that of DXPS) (Saravanan et al., 2017a). As for other TKs the reconstituted ChTK-F has a histidine residue at this position.

There are also some differences in the way that the TKs and DXPSs bind the cofactor TPP. While the residues and domains (PP- and Pyr-) that are used to bind TPP remain highly similar between the two enzymes the TK enzyme binds TPP between domains on two different monomers whereas DXPS binds TPP between domains on the same monomer.

**FIGURE 8:**
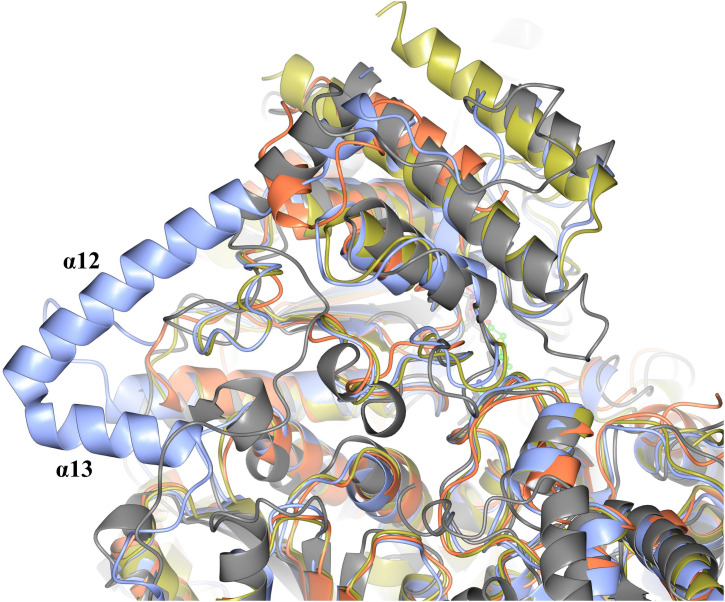
Structural superposition showing the cartoon representation of two helices (α12 and α13) that are located on the linker region between the PP and Pyr domains of EcTK (cyan) which are not in ChTK-F (purple) or the other TK related enzymes DXPS (gray) and PDH (yellow).

When comparing the structure of the ‘full-length’ TK enzymes with the ‘split-gene’, PDH and DXPS enzymes there is a linker (∼ 70 amino acids) that bridges the PP and Pyr domains that is not present in the ‘split-gene’ TKs, PDH and DXPS (Figure 8). One benefit of the ‘split-gene’ is that absence of the linker allows the enzyme to have ‘space’ between the two domains that could account for its ability to use the bulkier phenylacetaldehyde substrate demonstrated in this study.

## Conclusion

The individual ‘split-gene’ products of this novel TK enzyme have been shown to have no activity in either a tetrazolium red based colorimetric assay or the more accurate HPLC assay. This is not unexpected since the crystal structure of ChTK-F shows that the PP and Pyr domains lie on separate parts of the gene and both are required to bind the cofactor TPP which is essential for the reaction to take place. The ‘split-gene’ TKs are only present in archaea and some thermophilic bacteria (although then a full-length TK gene is also present) so *C. hydrogenoformans* is unique in being the only bacteria to possess only a ‘split-gene’ TK.

The ‘split-gene’ products of ChTK can be separately expressed and purified prior to being incubated together and further purified by size exclusion chromatography to yield an active transketolase enzyme (ChTK-F). This reconstituted enzyme has been shown to be active toward a broad range of bulkier aldehyde acceptors when used in the industrially important reaction using hydroxypyruvate as the ketol donor. The reconstituted enzyme has been biochemically characterized and has high thermal stability, is active at a range of pHs (pH 6–10, pH 8 optimum) and stable in 50% of a range of commonly used organic solvents.

The structure of the reconstituted enzyme reveals a heterotetrameric oligomeric state with two ChTK-N and two ChTK-C subunits forming the ChTK-F. The overall structure is similar to other full-length TK enzymes with TPP bound in the active site between the PP and Pyr domains. The major difference observed when the structures are compared is the absence of the linker between the PP and Pyr domains which could explain the ability of ChTK-F to turn over the bulkier substrates phenylacetaldehyde and cyclohexanecarboxaldehyde. The extensively studied EcTK is unable to use the same range of bulkier substrates which limits its synthetic range. Docking studies with ChTK-F involving phenylacetaldehyde have revealed a lysine (K33) residue that has the potential to help this substrate to be positioned during the reaction via a cation- π interaction with the positively charged lysine residue and the phenyl ring on the substrate (Supplementary Figure S3).

The high thermal stability of the ChTK-F enzyme can be explained by a combination of factors including the presence of the proline residue observed in other thermophilic TKs which when introduced into EcTK resulted in increased thermal stability of this mutant enzyme (Morris et al., 2016). The structure of the thermostable ChTK-F could help to further our understanding of thermostability of TK enzymes by predicting further mutations that could further increase the thermostability of mesophilic TKs. Further structural comparisons with the DXPS enzymes and ChTK-F could be used to predict mutations that will allow this enzyme to use a cheap substrate pyruvate as a ketol donor while keeping a broad acceptor substrate range to create high value chemicals.

The reconstituted active ‘split-gene’ TK enzyme ChTK-F has been shown in this study to have potential applications for industrial biocatalysis due to its ability to use bulkier aldehyde substrates and its overall stability to higher temperatures, a wider pH range and its tolerance to commonly used organic solvents.

## Materials and Methods

### Materials

All reagents were obtained from Sigma-Aldrich (Buchs, Switzerland) unless otherwise stated. The chromatography columns were obtained from GE Healthcare (Little Chalfont, United Kingdom). The expression vector (pLATE51) was obtained from Thermofisher Scientific, (Rochford, United Kingdom).

### Cloning, Expression, and Purification of ChTK-N and ChTK-C and Reconstitution of ChTK-F

The ‘split-genes’ were both cloned into the LIC site of pLATE51 vector and the expression carried out in *E. coli* BL21 DE3^∗^ cells. Cells were grown in 500 ml ZYM 5052 medium at 20°C and grown for 48 h. Cells were harvested by centrifugation (4700 x *g* at 18°C) and re-suspended in 50 mM Tris-HCl, pH 7.2, 2.4 mM TPP, 9 mM CaCl_2_, 0.5 mM NaCl, and 20 mM imidazole. The cells were disrupted by sonication at 10 μm (Soniprep150; MSE, London, United Kingdom) on ice for 4 min and the cell debris was removed by centrifugation at 20 000 x *g* at 4°C for 30 min. The protein was expressed in the soluble fraction and the clarified cell lysate was then heat-treated at 50°C for 30 min before being centrifuged at 20 000 *g* at 4°C for 30 min to remove any denatured proteins. The protein was purified using a 1 ml HisTrap FF crude column (GE Healthcare, Little Chalfont, United Kingdom) using a gradient from 20 to 500 mM imidazole in 50 mM Tris-HCl pH 7.2, 2.4 mM TPP, 9 mM CaCl_2_, 0.5 mM NaCl. The enzyme was then applied to a calibrated Superdex 200 HiLoad 16/60 gel filtration (GF) column (GE Healthcare, Little Chalfont, United Kingdom) and eluted with one column volume of 10 mM HEPES, 0.1 M NaCl, pH 7.2 at 1.0 ml min^–1^ with a yield of purified protein of 3.5 mg per liter of cell culture. Both individual enzymes (CHTK-N and CHTK-C) after GF were mixed in equimolar concentrations and left overnight at 4°C before being re-run on the calibrated Superdex 200 GF column.

### Colorimetric Assay

A reaction mixture (90 μl) containing propanal (50 mM), lithium hydroxypyruvate (LiHPA) (50 mM), TPP (2.4 mM), CaCl_2_ (9 mM) and TK sample (50% total volume – CHTK-C, CHTK-N and CHTK-F) in glycylglycine (50 mM, pH7.5) was incubated at 20°C for 17 hrs. 10 μl of the reaction mixture was then transferred to a micro-well containing MP-carbonate resin (Biotage AB) (10 mg) and glycylglycine buffer (90 μl, 50 mM, pH 7.0) and the mixture was incubated at 20°C for 3 hrs. 50 μl of this mixture (without the beads) was then diluted with further glycylglycine buffer (50 μl, 50 mM, pH 7.0), then tetrazolium red solution (20 μl, 0.2% 2,3,5 triphenyltetrazolium chloride in methanol) and finally 3 M NaOH (10 μl) with mixing.

### HPLC Assay

The enzyme kinetics was carried out using the HPLC assay that can be followed at 210 nm by the production of erythrulose or consumption of the ketol donor β-hydroxypyruvate (HPA). The cofactor solution (170 μl) (2 mM TPP, 9 mM CaCl_2_, 10 mM HEPES pH 7.2, 0.1 M NaCl) was added to the purified CHTK (N, C or F) sample (30 μl) and incubated for 20 mins at room temperature. The substrate solution (100 μl) (0–100 mM glycolaldehyde (GA), 0–100 mM LiHPA, 10 mM HEPES pH 7.2, 0.1 M NaCl) was added to start the reaction. Samples (20 μl) were taken at regular intervals, and the reaction quenched with 0.1% trifluoroacetic acid (TFA) in water (180 μl). Samples were applied to a Rezex RHM-Monosaccharide H^+^ (8%) (Phenomenex), using 0.1% TFA (mobile phase) 60°C and analyzed at 210 nm for LiHPA reduction and L/D-erythrulose production.

### Thermal Stability of ChTK-F

The thermostability of ChTK-F was investigated by incubating enzyme samples at a range of temperatures (20–90°C) for one hour using the gradient function in a SensOQuest LabCycler (Geneflow) before samples are cooled to 4°C and assayed for activity using the HPLC method described above.

### pH Stability of ChTK-F

The pH stability of ChTK-F was investigated by incubating the enzyme at room temperature for one hour in buffer solutions in the range of pH 5–12. The buffers used were 100 mM sodium acetate pH 5.0, 100 mM sodium phosphate pH 6.0, 100 mM Tris-HCl pH 7.0 – 9.0, 100 mM glycine-NaOH pH 10.0, 100 mM sodium dihydrogen orthophosphate-NaOH pH 11.0 – 12.0.

### Solvent Stability of ChTK

The residual activity of the ChTK-F was tested after incubation in a range of common organic solvents. The enzyme was incubated for 1 hour in buffer containing 25 mM Tris-HCl pH 7.5, 100 mM NaCl, and either 10%, 25% or 50% of methanol, ethanol, isopropanol, DMSO, acetonitrile and acetone. Samples were then assayed for activity using the HPLC method described above.

### Crystallization, Data Collection, and Structural Determination

The ChTK-F was concentrated to ∼10 mg ml^–1^ using a 10 kDa Vivaspin membrane (Vivaproducts, Littleton, Massachusetts, United States) and microbatch crystallization trials were set up using an Oryx 6 crystallization robot (Douglas Instruments, Hungerford, United Kingdom) using the JCSG+, PACT premier, MIDAS and Morpheus screens (Molecular Dimensions, Newmarket, United Kingdom; Newman et al., 2005). The droplet consisted of a 50:50 ratio of protein solution to screen solution and was covered with Al’s oil (a 50:50 mixture of silicone oil and paraffin) before being stored at 18°C and was regularly checked for growth of crystals using a light microscope.

The first crystals appeared in MIDAS-plus C10 (35% w/v polyacrylate 2100 sodium salt, 0.2 M ammonium sulfate, 0.1 M HEPES-NaOH pH7.5). The crystals were frozen in cryoprotectant containing 30% PEG 400 and indexed in two space groups P4_3_ and P4_3_2_1_2 (*a* = *b* = 92.4, *c* = 170.2 Å). Both crystal forms diffracted to 1.9Å, however the MR search for the ChTK-F components using MORDA molecular replacement pipeline (Vagin and Lebedev, 2015) could not position any ChTK-N in the crystal. Instead, four copies of the ChTK-C component were located in the P4_3_ crystal form (and 2 copies in the P4_3_2_1_2 crystal) and these crystals only contain the ChTK-C component. Both crystal forms were subject to preliminary refinement. The ChTK-C homo-tetramers formed were similar in both crystal forms and the P4_3_ form was chosen for further rebuilding on the basis of its better refinement statistics. The presence of sulfate ions in the crystallization conditions affected binding of the cofactor TPP resulting in the breakdown of ChTK-F. The crystallization of the protein in PEG-based conditions (0.1 MMT Buffer (malic acid/MES/Tris-HCl) pH 7.0, 25% w/v PEG 1500) resulted in crystals which diffracted to 2.1 Å and contained the intact CHTK-F heterotetramer with partial occupancy of TPP in the active site. An increase in the concentration of TPP in the crystallization conditions was found to prevent crystal growth.

However, when crystallized in the presence of DL-malic acid pH 7.0 and increased concentration of TPP [JCSG-plus F8 (2.1 M DL-malic acid pH 7.0)] better crystals were produced. The crystal was cryocooled in liquid N_2_ straight from the droplet. Data were collected on beamline I04-1 at the Diamond Synchrotron light source (Didcot, United Kingdom) at 100 K in a stream of gaseous nitrogen using a PILATUS detector. Data were processed in space group I222 (Table 1) and scaled using XDS (Kabsch, 2010) and AIMLESS (Evans and Murshudov, 2013) in the xia2 (Winter et al., 2013) pipeline. All further data and model manipulation were carried out using the CCP4 suite of programs (Winn et al., 2011). Phases for the CHTK-F crystal were determined using the molecular-replacement method as implemented in MOLREP (Vagin and Teplyakov, 2010) using preliminary refined CHTK-C and the N-terminal part of the EcTK model (PDB: 1QGD).

**TABLE 1:** Summary of crystallographic statistics.

Data collection statistics		
Crystal	ChTK β4	ChTK α2β2
Beamline	I04 DIAMOND	I04-1 DIAMOND
Wavelength (Å)	0.9795	0.9200
Space group	P43	I222
Unit Cell Parameters (Å)	*a* = *b* = 101.9, *c* = 164.6	*a* = 123.0, *b* = 130.0, *c* = 165.9
Total reflections^*a*^	687,440 (33,906)	1,762,697 (37,398)
Unique reflections^*a*^	131,289 (6,524)	288,149 (11,686)
Completeness (%)^*a*^	99.8 (99.8)	97.7 (81.3)
Rmeas (%)^*a,b*^	5.3 (424.5)	5.6 (161.9)
Mean I/σ^*a*^	13.1 (0.3)	16.4 (0.8)
Resolution range (Å)^*a*^	86.62 – 1.90 (1.93 –1.90)	60.80 – 1.34 (1.36 – 1.34)
CC1/2^*a,c*^	0.999 (0.339)	1.000 (0.266)
Wilson B-factor^*d*^ (Å2)	53.2	23.4
**Refinement Statistics**		
Rwork	0.178	0.141
Rfree	0.207	0.159
Number of non-hydrogen atoms	9,872	11,492
Macromolecules	9,436	9,864
Solvent	436	1,628
Protein residues	1,213	1,194
RMS bond lengths (Å)	0.008	0.007
RMS bond angles (°)	1.60	1.47
Ramachandran favored (%)^*e*^	96.48	97.37
Ramachandran outliers (%)^*e*^	0.42	0.0
Clashscore^*e*^	5.98	5.04
Average B-factor protein (Å2)	59.4	20.5
Average B-factor solvent (Å2)	67.2	38.2
PDB code	6YAJ	6YAK

*^*a*^Values for the highest resolution shell are given in parentheses. ^*b*^Rmeas =Σ*h*[*m*/(*m*−1)]1/2Σ|*iIh*,*i*− < *Ih* > |/Σ*h*Σ*iIh*,*i*. ^*c*^CC1/2 is defined in Karplus and Diederichs (2012). ^*d*^Wilson B-factor was estimated by SFCHECK (Vaguine et al., 1999). ^*e*^The Ramachandran statistics and clashscore statistics were calculated using MOLPROBITY (Williams et al., 2018).*

Electron-density maps were calculated and the structure was positioned to give the best fit to both the 2F_*o*_ – F_*c*_ and F_*o*_ – F_*c*_ maps. Maximum-likelihood refinement was performed using REFMAC 5 (Murshudov et al., 2011) after each session of model building performed in Coot (Emsley et al., 2010). Statistics of the data processing and the parameters of the final refined models are given in Table 1. The quality of the refined model was checked using PROCHECK (Laskowski et al., 1993) and MOLPROBITY (Williams et al., 2018). Images were created using the molecular-graphics programs PyMOL Molecular Graphics System, Version 2.0 Schrödinger, LLC and CCP4mg (McNicholas et al., 2011).

### Coordinates

The atomic coordinates and structure factors have been deposited in the Protein Data Bank as 6YAK (α_2_β_2_) and 6YAJ (β_4_).

### Computational Docking of Phenylacetaldehyde in the ChTK Active-Site

Phenylacetaldehyde was docked into the active site of TPP bound ChTK-F structure elucidated in this study (PDB: 6YAK) using Autodock 4.2 (Morris et al., 2009). The ligand phenylacetaldehyde was obtained from structure data files in the PDB (Phenylacetaldehyde: HY1) and the explorable space for docking was defined as a cube 10 Å in length centered at the carbanion on the thiazolium ring of TPP. Resulting docking solutions were studied using PyMOL.

## Data Availability Statement

The datasets presented in this study can be found in online repositories. The names of the repository/repositories and accession number(s) can be found below: http://www.wwpdb.org/, 6YAK and 6YAJ.

## Author Contributions

PJ identified, cloned, over-expressed, and crystallized the protein. The enzyme was biochemically characterized by PJ, IC, and SD. The structure was determined by PJ, CS, and MI. JL coordinated the project and wrote the manuscript with MI and PJ with additional contributions from all authors.

## Conflict of Interest

The authors declare that the research was conducted in the absence of any commercial or financial relationships that could be construed as a potential conflict of interest.

## References

[B1] Abdoul-Zabar J. Sorel I. Hélaine V. Charmantray F. Devamani T. Yi D. (2013). Thermostable transketolase from *Geobacillus stearothermophilus*: characterization and catalytic properties. *Adv. Synth. Catal.* 355 116–128. 10.1002/adsc.201200590

[B2] Arjunan P. Nemeria N. Brunskill A. Chandrasekhar K. Sax M. Yan Y. (2002). Structure of the pyruvate dehydrogenase multienzyme complex E1 component from *Escherichia coli* at 1.85 Å resolution. *Biochemistry* 41 5213–5221. 10.1021/bi0118557 11955070

[B3] Bawn M. Subrizi F. Lye G. J. Sheppard T. D. Hailes H. C. Ward J. M. (2018). One-pot, two-step transaminase and transketolase synthesis of l-gluco-heptulose from l-arabinose. *Enzyme Microb. Technol.* 116 16–22. 10.1016/j.enzmictec.2018.05.006 29887012

[B4] Bräsen C. Esser D. Rauch B. Siebers B. (2014). Carbohydrate metabolism in Archaea: current insights into unusual enzymes and pathways and their regulation. *Microbiol. Mol. Biol. Rev.* 78 89–175. 10.1128/MMBR.00041-13 24600042 PMC3957730

[B5] Costelloe S. J. Ward J. M. Dalby P. A. (2008). Evolutionary analysis of the TPP-dependent enzyme family. *J. Mol. Evol.* 66 36–49. 10.1007/s00239-007-9056-2 18043855

[B6] Dalby P. A. Aucamp J. P. George R. Martinez-Torres R. J. (2007). Structural stability of an enzyme biocatalyst. *Biochem. Soc. Trans.* 35 1606–1609. 10.1042/BST0351606 18031275

[B7] Egan K. Kelleher P. Field D. Rea M. C. Ross R. P. Cotter P. D. (2017). Genome Sequence of *Geobacillus stearothermophilus* DSM 458, an antimicrobial-producing thermophilic bacterium, isolated from a sugar beet factory. *Genome Announc.* 5:3809. 10.1128/genomeA.01172-17 29074660 PMC5658498

[B8] Emsley P. Lohkamp B. Scott W. G. Cowtan K. (2010). Features and development of Coot. *Acta Crystallogr. D. Biol. Crystallogr.* 66 486–501. 10.1107/S0907444910007493 20383002 PMC2852313

[B9] Evans P. R. Murshudov G. N. (2013). How good are my data and what is the resolution? *Acta Crystallogr. D. Biol. Crystallogr.* 69 1204–1214. 10.1107/S0907444913000061 23793146 PMC3689523

[B10] Fullam E. Pojer F. Bergfors T. Jones T. A. Cole S. T. (2012). Structure and function of the transketolase from *Mycobacterium tuberculosis* and comparison with the human enzyme. *Open Biol.* 2:110026. 10.1098/rsob.110026 22645655 PMC3352088

[B11] Gerhardt S. Echt S. Busch M. Freigang J. Auerbach G. Bader G. (2003). Structure and properties of an engineered transketolase from maize. *Plant Physiol.* 132 1941–1949. 10.1104/pp.103.020982 12913150 PMC181279

[B12] Hibbert E. G. Senussi T. Smith M. E. Costelloe S. J. Ward J. M. Hailes H. C. (2008). Directed evolution of transketolase substrate specificity towards an aliphatic aldehyde. *J. Biotechnol.* 134 240–245. 10.1016/j.jbiotec.2008.01.018 18342970

[B13] Hobbs G. R. Lilly M. D. Turner N. J. Ward J. M. Willets A. J. Woodley J. M. (1993). Enzyme-catalysed carbon–carbon bond formation: use of transketolase from *Escherichia coli*. *J. Chem. Soc. Perkin Trans.* 1 165–166. 10.1039/p19930000165

[B14] Jahromi R. R. Morris P. Martinez-Torres R. J. Dalby P. A. (2011). Structural stability of *E. coli* transketolase to temperature and pH denaturation. *J. Biotechnol.* 155 209–216. 10.1016/j.jbiotec.2011.06.023 21723889

[B15] Kabsch W. (2010). Xds. *Acta Crystallogr. D. Biol. Crystallogr.* 66 125–132. 10.1107/S0907444909047337 20124692 PMC2815665

[B16] Karplus P. A. Diederichs K. (2012). Linking crystallographic model and data quality. *Science* 336 1030–1033. 10.1126/science.1218231 22628654 PMC3457925

[B17] Kato M. Wynn R. M. Chuang J. L. Tso S. C. Machius M. Li J. (2008). Structural basis for inactivation of the human pyruvate dehydrogenase complex by phosphorylation: role of disordered phosphorylation loops. *Structure* 16 1849–1859. 10.1016/j.str.2008.10.010 19081061 PMC2849990

[B18] Koonin E. V. Galperin M. (2013). *Sequence — Evolution — Function.* Berlin: Springer Science & Business Media.

[B19] Kumar K. Woo S. M. Siu T. Cortopassi W. A. Duarte F. Paton R. S. (2018). Cation-pi interactions in protein-ligand binding: theory and data-mining reveal different roles for lysine and arginine. *Chem. Sci.* 9 2655–2665. 10.1039/c7sc04905f 29719674 PMC5903419

[B20] Laskowski R. A. MacArthur M. W. Moss D. S. Thornton J. M. (1993). PROCHECK: a program to check the stereochemical quality of protein structures. *J. Appl. Crystallogr.* 26 283–291. 10.1107/s0021889892009944

[B21] Leeper F. J. Hawksley D. Mann S. Perez Melero C. Wood M. D. (2005). Studies on thiamine diphosphate-dependent enzymes. *Biochem. Soc. Trans.* 33 772–775. 10.1042/BST0330772 16042596

[B22] Lilly M. D. Chauhan R. French C. Gyamerah M. Hobbs G. R. Humphrey A. (1996). Carbon-carbon bond synthesis: the impact of rDNA technology on the production and use of *E. coli* transketolase. *Ann. N.Y. Acad. Sci.* 782 513–525. 10.1111/j.1749-6632.1996.tb40589.x

[B23] Lindqvist Y. Schneider G. Ermler U. Sundström M. (1992). Three-dimensional structure of transketolase, a thiamine diphosphate dependent enzyme, at 2.5 A resolution. *EMBO J.* 11 2373–2379. 10.1002/j.1460-2075.1992.tb05301.x 1628611 PMC556711

[B24] Littlechild J. Novak H. James P. Sayer C. (2013). “Mechanisms of thermal stability adopted by thermophilic proteins and their use in white biotechnology,” in *Thermophilic Microbes in Environmental and Industrial Biotechnology: Biotechnology of Thermophiles*, eds Satyanarayana T. Littlechild J. Kawarabayasi Y. (Dordrecht: Springer Netherlands), 481–507. 10.1007/978-94-007-5899-5_19

[B25] Littlechild J. Turner N. Hobbs G. Lilly M. Rawas A. Watson H. (1995). Crystallization and preliminary X-ray crystallographic data with *Escherichia coli* transketolase. *Acta Crystallogr. D. Biol. Crystallogr.* 51 1074–1076. 10.1107/S0907444995005415 15299777

[B26] Littlechild J. A. Guy J. Connelly S. Mallett L. Waddell S. Rye C. A. (2007). Natural methods of protein stabilization: thermostable biocatalysts. *Biochem. Soc. Trans.* 35 1558–1563. 10.1042/BST0351558 18031266

[B27] Ludtke S. Neumann P. Erixon K. M. Leeper F. Kluger R. Ficner R. (2013). Sub-angstrom-resolution crystallography reveals physical distortions that enhance reactivity of a covalent enzymatic intermediate. *Nat. Chem.* 5 762–767. 10.1038/nchem.1728 23965678

[B28] Marsden S. R. Gjonaj L. Eustace S. J. Hanefeld U. (2017). Separating thermodynamics from Kinetics-A new understanding of the transketolase reaction. *ChemCatChem* 9 1808–1814. 10.1002/cctc.201601649 28919932 PMC5573996

[B29] Martin M. (2008). *Mechanistic Studies of Escherichia coli Transketolase.* Ph.D. thesis, University of Exeter, Exeter.

[B30] Martinez-Torres R. J. Aucamp J. P. George R. Dalby P. A. (2007). Structural stability of *E. coli* transketolase to urea denaturation. *Enzyme Microb. Technol.* 41 653–662. 10.1016/j.enzmictec.2007.05.019

[B31] McNicholas S. Potterton E. Wilson K. S. Noble M. E. (2011). Presenting your structures: the CCP4mg molecular-graphics software. *Acta Crystallogr. D. Biol. Crystallogr.* 67 386–394. 10.1107/S0907444911007281 21460457 PMC3069754

[B32] Mitschke L. Parthier C. Schroder-Tittmann K. Coy J. Ludtke S. Tittmann K. (2010). The crystal structure of human transketolase and new insights into its mode of action. *J. Biol. Chem.* 285 31559–31570. 10.1074/jbc.M110.149955 20667822 PMC2951230

[B33] Morris G. M. Huey R. Lindstrom W. Sanner M. F. Belew R. K. Goodsell D. S. (2009). AutoDock4 and AutoDockTools4: automated docking with selective receptor flexibility. *J. Comput. Chem.* 30 2785–2791. 10.1002/jcc.21256 19399780 PMC2760638

[B34] Morris K. G. Smith M. E. B. Turner N. J. Lilly M. D. Mitra R. K. Woodley J. M. (1996). Transketolase from *Escherichia coli*: a practical procedure for using the biocatalyst for asymmetric carbon-carbon bond synthesis. *Tetrahedron Asymmetr.* 7 2185–2188. 10.1016/0957-4166(96)00266-2

[B35] Morris P. Rios-Solis L. Garcia-Arrazola R. Lye G. J. Dalby P. A. (2016). Impact of cofactor-binding loop mutations on thermotolerance and activity of *E. coli* transketolase. *Enzyme Microb. Technol.* 89 85–91. 10.1016/j.enzmictec.2016.04.003 27233131

[B36] Muller Y. A. Lindqvist Y. Furey W. Schulz G. E. Jordan F. Schneider G. (1993). A thiamin diphosphate binding fold revealed by comparison of the crystal structures of transketolase, pyruvate oxidase and pyruvate decarboxylase. *Structure* 1 95–103. 10.1016/0969-2126(93)90025-c 8069629

[B37] Murshudov G. N. Skubak P. Lebedev A. A. Pannu N. S. Steiner R. A. Nicholls R. A. (2011). REFMAC5 for the refinement of macromolecular crystal structures. *Acta Crystallogr. D. Biol. Crystallogr.* 67 355–367. 10.1107/S0907444911001314 21460454 PMC3069751

[B38] Newman J. Egan D. Walter T. S. Meged R. Berry I. Ben Jelloul M. (2005). Towards rationalization of crystallization screening for small- to medium-sized academic laboratories: the PACT/JCSG+ strategy. *Acta Crystallogr. D. Biol. Crystallogr.* 61 1426–1431. 10.1107/S0907444905024984 16204897

[B39] Nikkola M. Lindqvist Y. Schneider G. (1994). Refined structure of transketolase from *Saccharomyces cerevisiae* at 2.0 A resolution. *J. Mol. Biol.* 238 387–404. 10.1006/jmbi.1994.1299 8176731

[B40] Payongsri P. Steadman D. Hailes H. C. Dalby P. A. (2015). Second generation engineering of transketolase for polar aromatic aldehyde substrates. *Enzyme Microb. Technol.* 71 45–52. 10.1016/j.enzmictec.2015.01.008 25765309

[B41] Payongsri P. Steadman D. Strafford J. MacMurray A. Hailes H. C. Dalby P. A. (2012). Rational substrate and enzyme engineering of transketolase for aromatics. *Org. Biomol. Chem.* 10 9021–9029. 10.1039/c2ob25751c 23079923

[B42] Racker E. De La Haba G. Leder I. G. (1954). Transketolase-catalyzed utilization of fructose 6-phosphate and its significance in a glucose 6-phosphate oxidation cycle. *Arch. Biochem. Biophys.* 48 238–240. 10.1016/0003-9861(54)90331-8 13125597

[B43] Rodionova I. A. Yang C. Li X. Kurnasov O. V. Best A. A. Osterman A. L. (2012). Diversity and versatility of the *Thermotoga maritima* sugar kinome. *J. Bacteriol.* 194 5552–5563. 10.1128/JB.01136-12 22885293 PMC3458674

[B44] Saravanan T. Junker S. Kickstein M. Hein S. Link M. K. Ranglack J. (2017a). Donor promiscuity of a thermostable transketolase by directed evolution: efficient complementation of 1-Deoxy-d-xylulose-5-phosphate synthase activity. *Angew. Chem. Int. Ed. Engl.* 56 5358–5362. 10.1002/anie.201701169 28378514

[B45] Saravanan T. Reif M.-L. Yi D. Lorillière M. Charmantray F. Hecquet L. (2017b). Engineering a thermostable transketolase for arylated substrates. *Green Chemistry* 19 481–489. 10.1039/c6gc02017h

[B46] Schenk G. Duggleby R. G. Nixon P. F. (1998). Properties and functions of the thiamin diphosphate dependent enzyme transketolase. *Int. J. Biochem. Cell Biol.* 30 1297–1318. 10.1016/s1357-2725(98)00095-8 9924800

[B47] Selivanov V. Kovina M. Kochevova V. Meshalkina L. Kochetov A. (2003). Studies of thiamin diphosphate binding to the yeast apotransketolase. *J. Mol. Catal. B Enzym* 26 33–40. 10.1016/S1381-1177(03)00115-2

[B48] She Q. Singh R. K. Confalonieri F. Zivanovic Y. Allard G. Awayez M. J. (2001). The complete genome of the crenarchaeon *Sulfolobus solfataricus* P2. *Proc. Natl. Acad. Sci. U.S.A.* 98 7835–7840. 10.1073/pnas.141222098 11427726 PMC35428

[B49] Smith M. E. Kaulmann U. Ward J. M. Hailes H. C. (2006). A colorimetric assay for screening transketolase activity. *Bioorg. Med. Chem.* 14 7062–7065. 10.1016/j.bmc.2006.06.008 16784864

[B50] Soderberg T. (2005). Biosynthesis of ribose-5-phosphate and erythrose-4-phosphate in archaea: a phylogenetic analysis of archaeal genomes. *Archaea* 1 347–352. 10.1155/2005/314760 15876568 PMC2685555

[B51] Studier F. W. (2005). Protein production by auto-induction in high density shaking cultures. *Protein Expr. Purif.* 41 207–234. 10.1016/j.pep.2005.01.016 15915565

[B52] Suzuki Y. Oishi K. Nakano H. Nagayama T. (1987). A strong correlation between the increase in number of proline residues and the rise in thermostability of five Bacillus oligo-1,6-glucosidases. *Appl. Microbiol. Biot.* 26 546–551. 10.1007/bf00253030

[B53] Svetlichny V. A. Sokolova T. G. Gerhardt M. Ringpfeil M. Kostrikina N. A. Zavarzin G. A. (1991). *Carboxydothermus hydrogenoformans* gen. nov., sp. nov., a CO-utilizing Thermophilic anaerobic bacterium from hydrothermal environments of Kunashir Island. *Syst. Appl. Microbiol.* 14 254–260. 10.1016/S0723-2020(11)80377-2

[B54] Vagin A. Lebedev A. (2015). MoRDa, an automatic molecular replacement pipeline. *Acta Crystallogr. Section A Foundations Adv.* 71:s19 10.1107/s2053273315099672

[B55] Vagin A. Teplyakov A. (2010). Molecular replacement with MOLREP. *Acta Crystallogr. D. Biol. Crystallogr.* 66 22–25. 10.1107/S0907444909042589 20057045

[B56] Vaguine A. A. Richelle J. Wodak S. J. (1999). SFCHECK: a unified set of procedures for evaluating the quality of macromolecular structure-factor data and their agreement with the atomic model. *Acta Crystallogr. D. Biol. Crystallogr.* 55 191–205. 10.1107/S0907444998006684 10089410

[B57] Veitch N. J. Maugeri D. A. Cazzulo J. J. Lindqvist Y. Barrett M. P. (2004). Transketolase from *Leishmania mexicana* has a dual subcellular localization. *Biochem. J.* 382 759–767. 10.1042/BJ20040459 15149284 PMC1133835

[B58] Waters E. Hohn M. J. Ahel I. Graham D. E. Adams M. D. Barnstead M. (2003). The genome of *Nanoarchaeum equitans*: insights into early archaeal evolution and derived parasitism. *Proc. Natl. Acad. Sci. U.S.A.* 100 12984–12988. 10.1073/pnas.1735403100 14566062 PMC240731

[B59] Williams C. J. Headd J. J. Moriarty N. W. Prisant M. G. Videau L. L. Deis L. N. (2018). MolProbity: more and better reference data for improved all-atom structure validation. *Protein Sci.* 27 293–315. 10.1002/pro.3330 29067766 PMC5734394

[B60] Winn M. D. Ballard C. C. Cowtan K. D. Dodson E. J. Emsley P. Evans P. R. (2011). Overview of the CCP4 suite and current developments. *Acta Crystallogr. D. Biol. Crystallogr.* 67 235–242. 10.1107/S0907444910045749 21460441 PMC3069738

[B61] Winter G. Lobley C. M. Prince S. M. (2013). Decision making in xia2. *Acta Crystallogr. D. Biol. Crystallogr.* 69 1260–1273. 10.1107/S0907444913015308 23793152 PMC3689529

[B62] Wu M. Ren Q. Durkin A. S. Daugherty S. C. Brinkac L. M. Dodson R. J. (2005). Life in hot carbon monoxide: the complete genome sequence of *Carboxydothermus hydrogenoformans* Z-2901. *PLoS Genet.* 1:e65. 10.1371/journal.pgen.0010065 16311624 PMC1287953

[B63] Xiang S. Usunow G. Lange G. Busch M. Tong L. (2007). Crystal structure of 1-deoxy-D-xylulose 5-phosphate synthase, a crucial enzyme for isoprenoids biosynthesis. *J. Biol. Chem.* 282 2676–2682. 10.1074/jbc.M610235200 17135236

[B64] Yi D. Devamani T. Abdoul-Zabar J. Charmantray F. Helaine V. Hecquet L. (2012). A pH-based high-throughput assay for transketolase: fingerprinting of substrate tolerance and quantitative kinetics. *Chembiochem* 13 2290–2300. 10.1002/cbic.201200364 23001740

