# Supplementary material for: A ‘Split-Gene’ Transketolase From the Hyper-Thermophilic Bacterium Carboxydothermus hydrogenoformans: Structure and Biochemical Characterization
Source: Front Microbiol. 2020 Oct 30;11:592353. doi: 10.3389/fmicb.2020.592353 (PMC7661550; doi:10.3389/fmicb.2020.592353)
Supplement: Supplementary file 1 [file Image_1.PDF]

Supplementary Material

Supplementary Figures

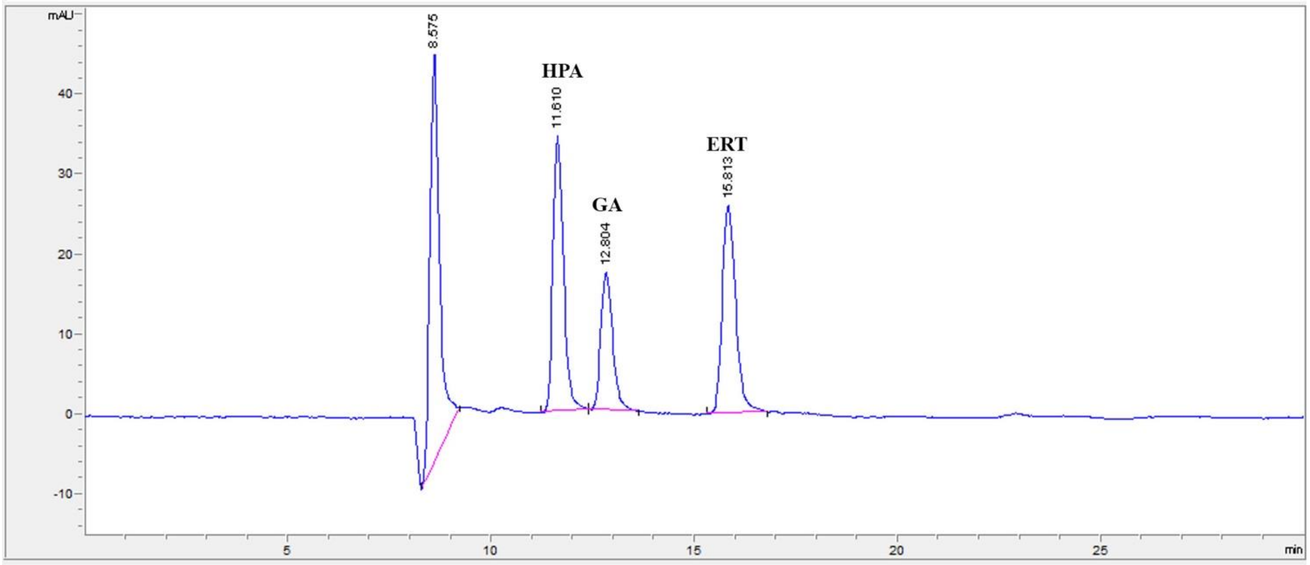

Figure S1

Standard HPLC trace showing the production of erythrulose (ERT) from the substrates glycolaldehyde (GA) and hydroxypyruvate (HPA) from the ChTK-F enzymatic reaction

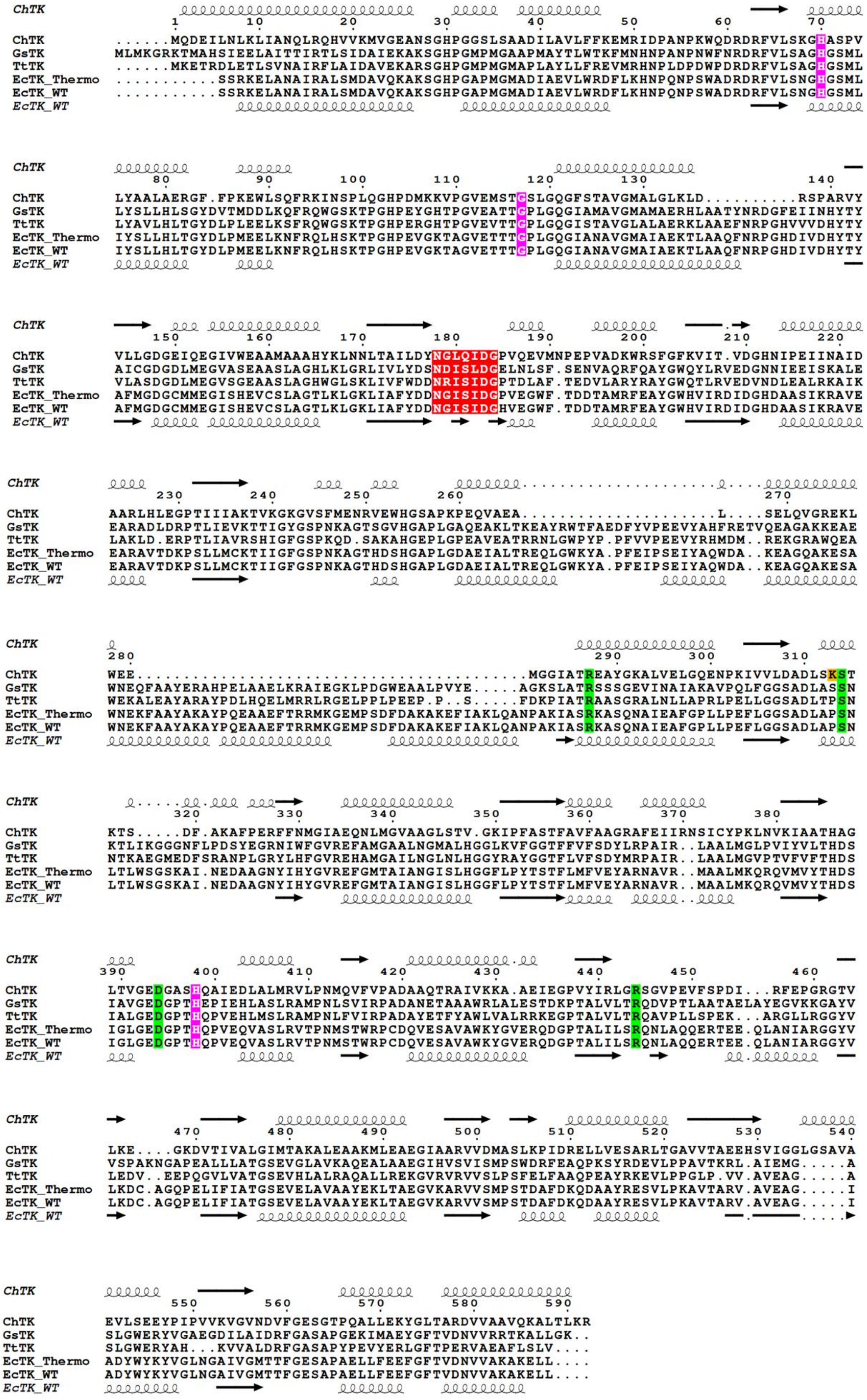
ChTK
EcTK_WT
1 10 20 30 40 50 60 70
ChTK ......MQDEILNLKLIANQLRQHVVKMVGEANSGHPGGSLSAADILAVLFFKEMRIDPANPKWQDRDRFVLSKGHASPV
GsTK MLMKGRKTMAHSIEELAITTIRTLSIDAIEKAKSGHPGMPMGAAPMAYTLWTKFMNHNPANPNWFNRDRFVLSAGHGSML
TtTK ......MKETRDLETLSVNAIRFLAIDAVEKARSGHPGMPMGMAPLAYLLFREVMRHNPLDPDWPDRDRFVLSAGHGSML
EcTK_Thermo ...........SSRKELANAIRALSMDAVQKAKSGHPGAPMGMADIAEVLWRDFLKHNPQNPSWADRDRFVLSNGHGSML
EcTK_WT ...........SSRKELANAIRALSMDAVQKAKSGHPGAPMGMADIAEVLWRDFLKHNPQNPSWADRDRFVLSNGHGSML
80 90 100 110 120 130 140
ChTK LYAALAERGF.FPKEWLSQFRKINSPLQGHPDMKKVPGVEMSTGSLGQGFSTAVGMALGLKLD..........RSPARVY
GsTK LYSLLHLSGYDVTMDDLKQFRQWGSKTPGHPEYGHTPGVEATTGPLGQGIAMAVGMAMAERHLAATYNRDGFEIINHYTY
TtTK LYAVLHLTGYDLPLEELKSFRQWGSKTPGHPERGHTPGVEVTTGPLGQGISTAVGLALAERKLAAEFNRPGHVVVDHYTY
EcTK_Thermo IYSLLHLTGYDLPMEELKNFRQLHSKTPGHPEVGKTAGVETTTGPLGQGIANAVGMAIAEKTLAAQFNRPGHDIVDHYTY
EcTK_WT IYSLLHLTGYDLPMEELKNFRQLHSKTPGHPEVGKTAGVETTTGPLGQGIANAVGMAIAEKTLAAQFNRPGHDIVDHYTY
150 160 170 180 190 200 210 220
ChTK VLLGDGEIQEGIVWEAAMAAAHYKLNNLTAILDYNGLQIDGPVQEVMNPEPVADKWRSFGFKVIT.VDGHNIPEIINAID
GsTK AICGDGDLMEGVASEAASLAGHLKLGRLIVLYDSNDISLDGELNLSF.SENVAQRFQAYGWQYLRVEDGNNIEEISKALE
TtTK VLASDGDLMEGVSGEAASLAGHWGLSKLIVFWDDNRISIDGPTDLAF.TEDVLARYRAYGWQTLRVEDVNDLEALRKAIK
EcTK_Thermo AFMGDGCMMEGISHEVCSLAGTLKLGKLIAFYDDNGISIDGPVEGWF.TDDTAMRFEAYGWHVIRDIDGHDAASIKRAVE
EcTK_WT AFMGDGCMMEGISHEVCSLAGTLKLGKLIAFYDDNGISIDGHVEGWF.TDDTAMRFEAYGWHVIRDIDGHDAASIKRAVE
230 240 250 260 270
ChTK AARLHLEGPTIIIAKTVKGKGVSFMENRVEWHGSAPKPEQVAEA.....................L....SELQVGREKL
GsTK EARADLDRPTLIEVKTTIGYGSPNKAGTSGVHGAPLGAQEAKLTKEAYRWTFAEDFYVPEEVYAHFRETVQEAGAKKEAE
TtTK LAKLD.ERPTLIAVRSHIGFGSPKQD.SAKAHGEPLGPEAVEATRRNLGWPYP.PFVVPEEVYRHMDM..REKGRAWQEA
EcTK_Thermo EARAVTDKPSLLMCKTIIGFGSPNKAGTHDSHGAPLGDAEIALTREQLGWKYA.PFEIPSEIYAQWDA..KEAGQAKESA
EcTK_WT EARAVTDKPSLLMCKTIIGFGSPNKAGTHDSHGAPLGDAEIALTREQLGWKYA.PFEIPSEIYAQWDA..KEAGQAKESA
280 290 300 310
ChTK WEE...MGGIATREAYGKALVELGQENPKIVVLDADLSKST
GsTK WNEQFAAYERAHPELAAELKRAIEGKLPDGWEEALPVYE.....AGKSLATRSSSGEVINAIAKAVPQLFGGSADLASSN
TtTK WEKALEAYARAYPDLHQELMRRLRGELPPLPEEP.P..S.....FDKPIATRAASGRALNLLAPRLPELLGGSADLTPSN
EcTK_Thermo WNEKFAAYAKAYPQEAAEFTRRMKGEMPSDFDAKAKEFIAKLQANPAKIASRKASQNAIEAFGPLLPEFLGGSADLAPSN
EcTK_WT WNEKFAAYAKAYPQEAAEFTRRMKGEMPSDFDAKAKEFIAKLQANPAKIASRKASQNAIEAFGPLLPEFLGGSADLAPSN
320 330 340 350 360 370 380
ChTK KTS.....DF.AKAFPERFFNMGIAEQNLMGVAAGLSTV.GKIPFASTFAVFAAGRAFEIIRNSICYPKLNVKIAATHAG
GsTK KTLIKGGGNFLPDSYEGRNIWFGVREFAMGAALNGMALHGGLKVFGGTFFVFSDYLRPAIR..LAALMGLPVIYVLTHDS
TtTK NTKAEGMEDFSRANPLGRYLHFGVREHAMGAILNGLNLHGGYRAYGGTFLVFSDYMRPAIR..LAALMGVPTVFVFTHDS
EcTK_Thermo LTLWSGSKAI.NEDAAGNYIHYGVREFGMTAIANGISLHGGFLPYTSTFLMFVEYARNAVR..MAALMKQRQVMVYTHDS
EcTK_WT LTLWSGSKAI.NEDAAGNYIHYGVREFGMTAIANGISLHGGFLPYTSTFLMFVEYARNAVR..MAALMKQRQVMVYTHDS
390 400 410 420 430 440 450 460
ChTK LTVGEDGASHQAIEDLALMRVLPNMQVFVPADAAQTRAIVKKA.AEIEGPVYIRLGRSGVPEVFSPDI...RFEPGRGTV
GsTK IAVGEDGPTHEPIEHLASLRAMPNLSVIRPADANETAAAWRLALESTDKPTALVLTRQDVPTLAATAELAYEGVKKGAYV
TtTK IALGEDGPTHQPVEHLMSLRAMPNLFVIRPADAYETFYAWLVALRRKEGPTALVLTRQAVPLLSPEK...ARGLLRGGYV
EcTK_Thermo IGLGEDGPTHQPVEQVASLRVTPNMSTWRPCDQVESAVAWKYGVERQDGPTALILSRQNLAQQERTEE.QLANIARGGYV
EcTK_WT IGLGEDGPTHQPVEQVASLRVTPNMSTWRPCDQVESAVAWKYGVERQDGPTALILSRQNLAQQERTEE.QLANIARGGYV
470 480 490 500 510 520 530 540
ChTK LKE....GKDVTIVALGIMTAKALEAAKMLEAEGIAARVVDMASLKPIDRELLVESARLTGAVVTAEEHSVIGGLGSAVA
GsTK VSPAKNGAPEALLLATGSEVGLAVKAQEALAAEGIHVSVISMPSWDRFEAQPKSYRDEVLPPAVTKRL.AIEMG.....A
TtTK LEDV..EEPQGVLVATGSEVHLALRAQALLREKGVRVRVVSLPSFELFAAQPEAYRKEVLPPGLP.VV.AVEAG.....A
EcTK_Thermo LKDC.AGQPELIFIATGSEVELAVAAYEKLTAEGVKARVVSMPSTDAFDKQDAAYRESVLPKAVTARV.AVEAG.....I
EcTK_WT LKDC.AGQPELIFIATGSEVELAVAAYEKLTAEGVKARVVSMPSTDAFDKQDAAYRESVLPKAVTARV.AVEAG.....I
550 560 570 580 590
ChTK EVLSEEYPIPVVKVGVNDVFGESGTPQALLEKYGLTARDVVAAVQKALTLKR
GsTK SLGWERYVGAEGDILAIDRFGASAPGEKIMAEYGFTVDNVVRRTKALLGK..
TtTK SLGWERYAH...KVVALDRFGASAPYPEVYERLGFTPERVAEAFLSLV....
EcTK_Thermo ADYWYKYVGLNGAIVGMTTFGESAPAELLFEEFGFTVDNVVAKAKELL....
EcTK_WT ADYWYKYVGLNGAIVGMTTFGESAPAELLFEEFGFTVDNVVAKAKELL....

Figure S2

Sequence alignment of six TK enzymes mentioned in this study (ChTK-F, GsTK, TtTK, EcTK_wt and EcTK_Thermo). Secondary structure elements from ChTK-F are shown above and secondary structure elements from EcTK are shown below. The residues involved in aromatic substrate binding (green box), donor substrate binding (purple box), important for thermal stability (red box) and potential stabilising from the docking experiment (orange box) are indicated. The figure was created with ESPript using the ENDscript server (Robert *et al*., 2014).

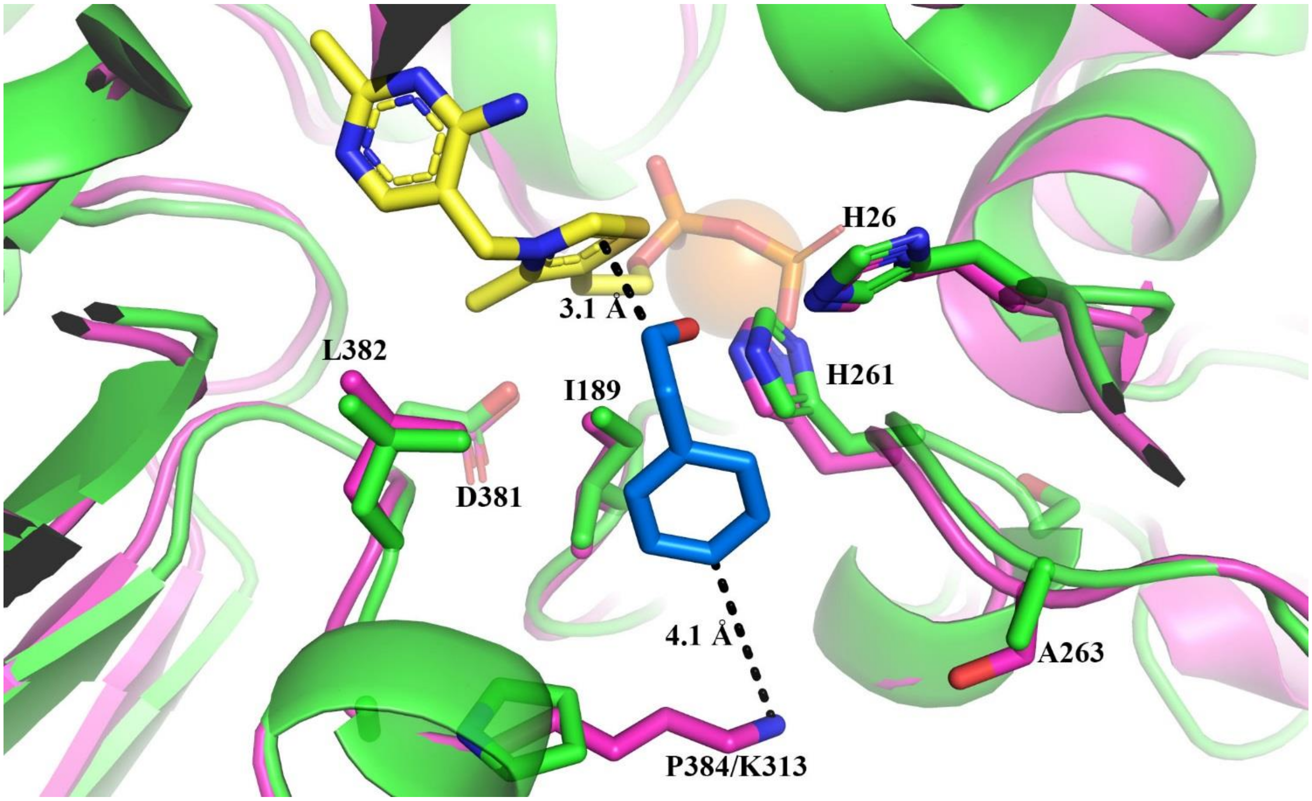

Figure S3.

Computational docking of phenylacetaldehyde (blue) into the active site of ChTK-F (pink) with EcTK (green) superimposed. This shows the relative position of the carbanion on TPP (yellow) to the carbonyl on phenylacetaldehyde so that it may undergo nucleophilic attack. The position of this substrate could be further stabilised by a cation- π interaction between the aromatic ring of phenylacetaldehyde and a lysine residue (K33) present in ChTK-F but not in EcTK. Amino acid side chains of those residues involved in substrate binding are shown for comparison. The Ca^{2+} ion is shown as an orange sphere. Figures S3 and S4 were prepared using the PyMOL Molecular Graphics System Version 2 Schrodinger LLC.

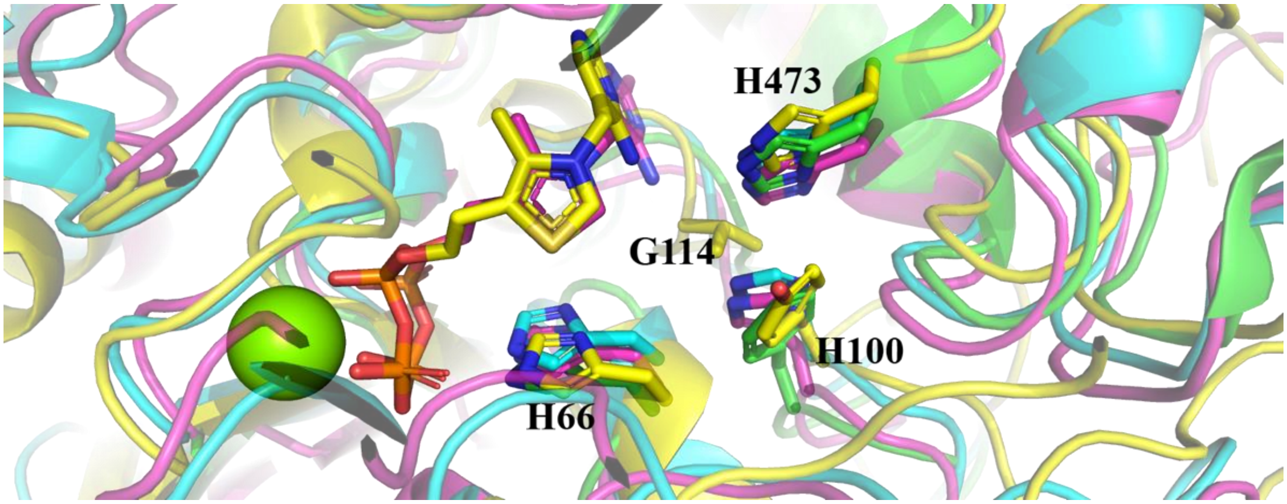

Figure S4.
A cartoon model showing the structural alignment of the residues associated with the donor substrate binding between ChTK-F (purple), EcTK, (cyan) EcDXPS (yellow) and EcPDH (green). The amino acid side chains responsible for substrate binding are labelled and numbered according to the EcTK sequence. The TPP molecule is shown as a stick model. The Ca^{2+} ion is shown as a green sphere.

**Substrate**	**Structure**	**ChTK-F Activity**
Glycolaldehyde	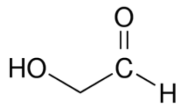	Active (++)
Phenylacetaldehyde	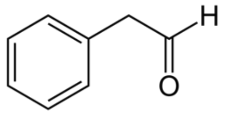	Active (+)
Cyclohexanecarboxaldehyde	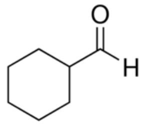	Active (+)

Figure S5
A) The aldehyde acceptors shown to be active with the reaction of CHTK-F together with hydroxypyruvate (HPA). The enzyme was more active towards glycolaldehyde (++) as the acceptor but also showed activity towards phenylacetaldehyde (+) and cyclohexanecarboxaldehyde (+).

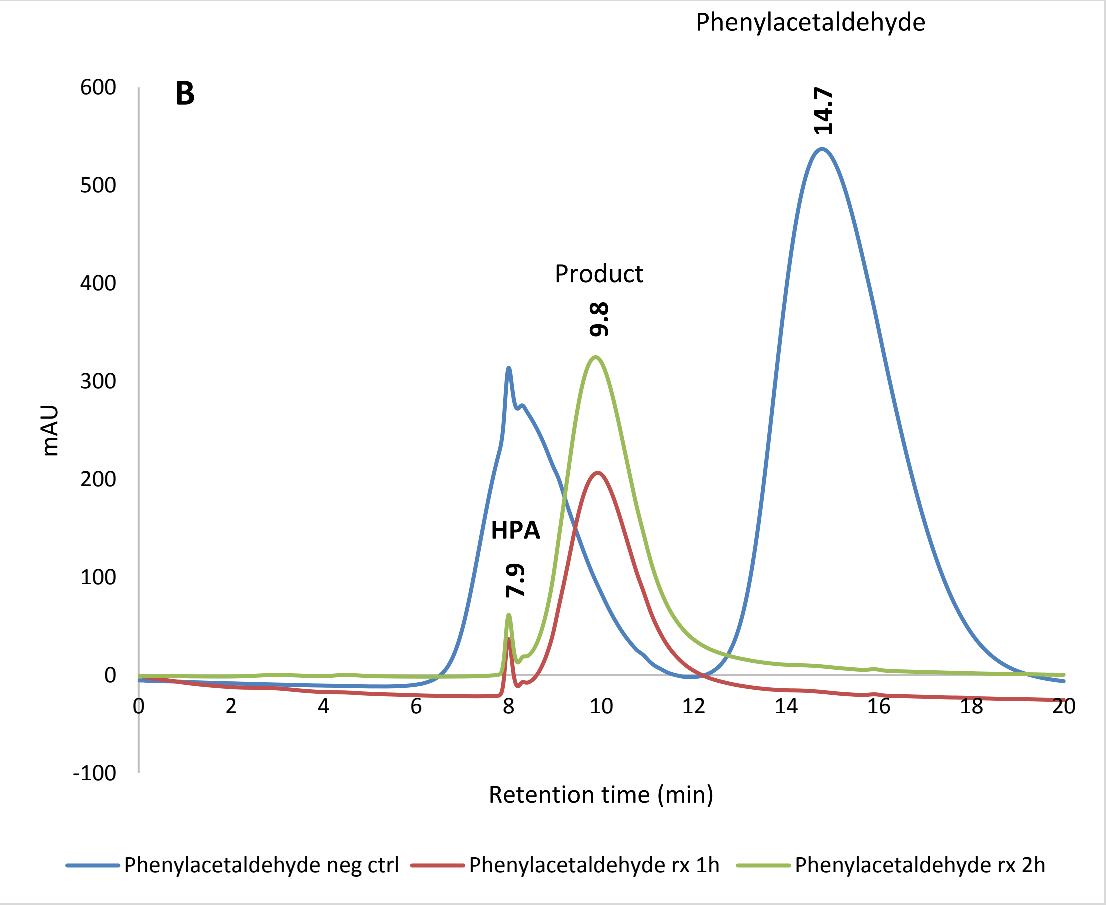

B) Standard HPLC trace showing the reaction of CHTK-F with the substrates phenylacetaldehyde and hydroxypyruvate (HPA). The control reaction is shown in blue and the reactions after 1 hour and

2 hour incubation are shown in red and green respectively. The presence of a peak at 9.8 min is attributed to the product 1,3-dihydroxy-4-phenyl-2-butanone formation.

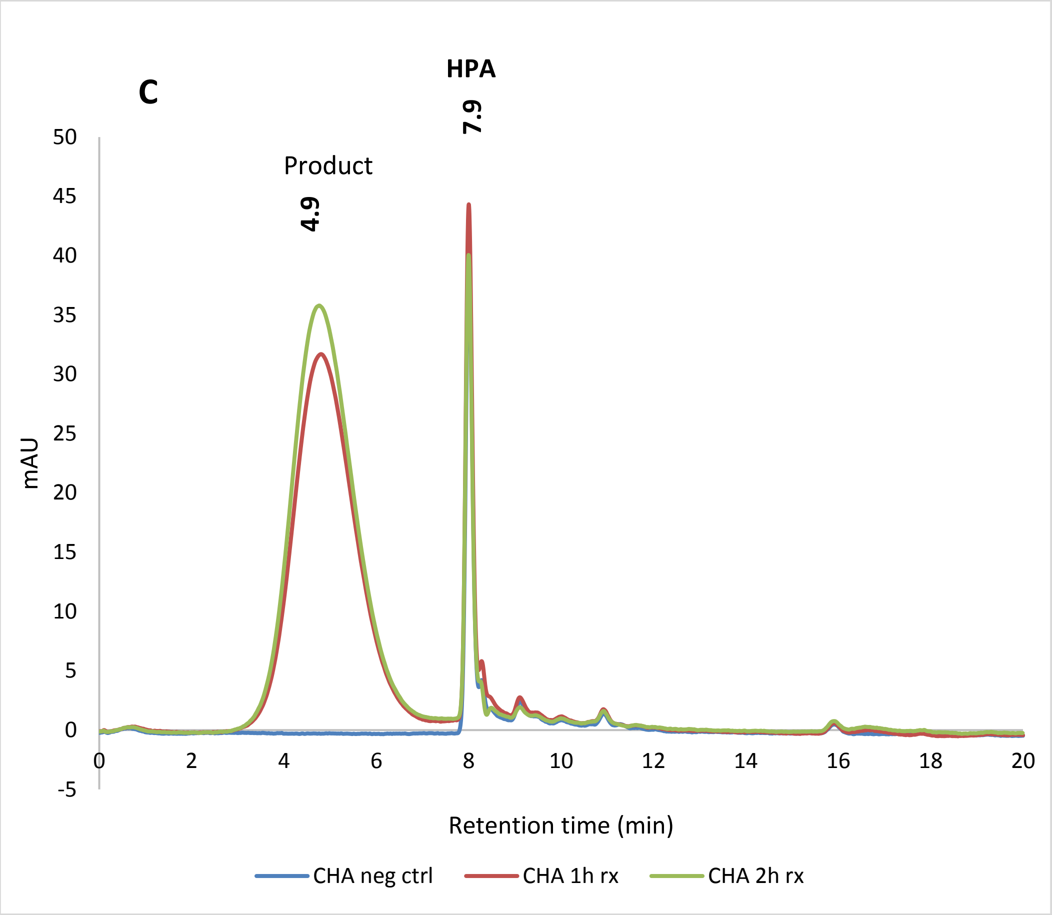

C) Standard HPLC trace showing the showing the reaction of CHTK-F with the substrates cyclohexanecarboxaldehyde and hydroxypyruvate (HPA). The control reaction is shown in blue and 1 hour and 2 hour incubation reactions are shown in red and green respectively**.** The presence of a peak at 4.9 min is attributed to the product 1-cyclohexyl-1,3-dihydroxyacetone formation.

References

Robert, X., and Gouet, P. (2014). Deciphering key features in protein structures with the new ENDscript server. *Nucleic Acids Res.* 42, 320–324. doi: 10.1093/nar/gku316
